# Airway Management in the Emergency Department (The OcEAN-Study) - a prospective single centre observational cohort study

**DOI:** 10.1186/s13049-019-0599-1

**Published:** 2019-02-14

**Authors:** Michael Bernhard, Sönke Nils Bax, Thomas Hartwig, Maryam Yahiaoui-Doktor, Sirak Petros, Sven Bercker, Alexandra Ramshorn-Zimmer, André Gries

**Affiliations:** 1 Emergency Department, University Hospital of Düsseldorf, Düsseldorf, Germany; 2 Emergency Department, University Hospital of Leipzig, Leipzig, Germany; 3 Working group “Trauma and Resuscitation Room Management“, Task Force Emergency Medicine, German Society of Anaesthesiology and Intensiv care Medizin, Nürnberg, Germany; 4 Institute for Medical Informatics, Statistics and Epidemiology (IMISE), University of Leipzig, Leipzig, Germany; 5 Medical Intensive Care Unit, University Hospital of Leipzig, Leipzig, Germany; 6 Department of Anaesthesiology and Intensive Care Medicine, University Hospital of Leipzig, Leipzig, Germany; 7 Emergency Department, Paracelsus Hospital of Henstedt-Ulzburg, Wilstedter Straße 134, D-24558 Henstedt-Ulzburg, Germany

**Keywords:** Airway management, Emergency department, Resuscitation room, First-pass success, Complications

## Abstract

**Background:**

Emergency airway management (AM) is a major key for successful resuscitation of critically ill non-traumatic (CINT) patients. Details of the AM of these patients in German emergency departments (ED) are unknown. This observational study describes epidemiology, airway techniques, success rates and complications of AM in CINT ED patients in the resuscitation room (RR).

**Methods:**

Data was collected prospectively on adult CINT patients admitted to the RR of a single German university ED September 2014 to August 2015. Patient characteristics, out-of-hospital and in-hospital RR AM, complications and success rates were recorded using a self-developed airway registry form.

**Results:**

During the study period 34,303 patients were admitted to the ED, out of those 21,074 patients for non-trauma emergencies. Suffering from severe acute life-threatening problems, 532 CINT patients were admitted to the RR. 150 (28.2%) CINT patients had received out-of-hospital AM. In 16 of these cases (10.7%) the inserted airway needed to be changed after RR admission (unrecognized oesophageal intubation: *n* = 2, laryngeal tube exchange: *n* = 14). 136 (25.6%) CINT patients without out-of-hospital AM received RR AM immediately after admission. The first-pass and overall success rate in the RR were 71 and 100%, respectively, and multiple intubation attempts were necessary in 29%. A lower Cormack/Lehane (C/L) grade was associated with less intubation attempts (C/L1/2 vs. 3/4: 1.2 ± 0.5 vs. 1.8 ± 1.2, *p* = 0.0002). Complication rate was 43%.

**Conclusions:**

OcEAN demonstrates the challenges of AM in CINT patients in a German ED RR. We propose a nation-wide ED airway registry to better track outcomes in the future.

**Electronic supplementary material:**

The online version of this article (10.1186/s13049-019-0599-1) contains supplementary material, which is available to authorized users.

## Background

Critically ill patients frequently require airway management in the field or in the Emergency Department (ED) [1]. Several investigations have shown that emergency airway management in the field and in the ED is associated with adverse events and complications (e.g., hypoxemia, oesophageal intubation, hypotension) [2, 3]. However, inadequate oxygenation and ventilation will lead to wrong outcome and therefore emergency airway management is of priority in resuscitation of critically ill patients [4, 5].

Studies have demonstrated that the number of intubation attempts is associated with increasing complication rates, therefore, the “first-pass intubation success” is an important concept in emergency airway management [6, 7]. ED Airway registries exist in some countries (e.g., Australia [8], North America [9, 10], Korea [11], Japan [12]), however data on emergency airway management in German EDs are still missing.

The aim of this study is to evaluate airway management in critically ill patients in the resuscitation room (RR) of a German ED in order to describe incidence, devices, techniques, success and complication rates.

## Methods

### Study design

This prospective single centre observational cohort [Observation of airway management in Emergency Department (OcEAN)] study was carried out from 1 September 2014 to 31 August 2015 in the ED of the University Hospital of Leipzig, Germany. The OcEAN study was approved by the ethical committee of the Medical Faculty of the University of Leipzig, Germany (265–14-25,082,014).

### Setting

More than 34,000 patients are managed annually in the ED of the University Hospital of Leipzig, a level 1 trauma centre. However, about 50% of patients suffering from non-traumatic acute problems or emergencies. The out-of-hospital emergency care is provided by an EMS system staffed with paramedics and EMS physicians. In our institution, all non-traumatic critically ill patients in the RR are treated by a team of two nurses, one resident and one senior physician with emergency and intensive care competency. Patients fulfilling the non-trauma RR activation criteria according to Additional file 1: Table S1 (in the Supplemental material) are admitted to the RR, the others are treated in other regions of the ED as the observation unit or one of the single cabins.

### Study definition and data collection

All adult non-traumatic critically ill patients needing airway management in the ED RR were consecutively included. Paediatric and trauma patients were excluded. For further analysis, data were documented in a self-developed and implemented airway registry form. The airway registry form included the “Utstein airway core variables” established in the out-of-hospital airway management, as well as parameters implemented in out-of-hospital and ED airway registries in North America and Austria, as well as other out-of-hospital studies from Germany [4, 5, 8, 9, 13–16].

The OcEAN airway registry form was completed in the RR, any missing data were followed up through interviews with the staff involved or from the medical records.

The OcEAN airway registry form included the patient’s characteristics (age, gender, weight, high, body mass index), out-of-hospital triage score using American Society of Anesthesiology (ASA) score [17] at hospital admission and National Advisory Committee of Aeronautics (NACA) score in order to stratify the patient cohort [18], as well as the chief complaint leading to ED admission [cardiac arrest, unconsciousness (Glasgow coma scale [19] < 9), respiratory failure, shock].

The out-of-hospital airway management records were reviewed by the main investigator [airway management technique performed by EMS physicians including endotracheal intubation, supraglottic airway device (SAD), cricothyroidotomy, success of airway management, use of capnography].

The ED airway management was recorded, including patient position [back-up head elevated (BUHE [20]) or supine position], immobilization, and airway device [Macintosh blade, video laryngoscope, SAD (laryngeal tube, laryngeal mask airway), cricothyroidotomy, tracheotomy tube]. The number of intubation attempts per patient was also recorded. An airway management attempt was defined as the insertion of the airway device in the mouth (i.e., single passage of a laryngoscopy blade behind the lips, insertion of SAD). Multiple intubation attempts were defined as more than one insertion attempt. Per our institutional safety protocol, physicians had to handover the airway procedure to another physician after a second failed attempt at airway management. Difficult airway characteristics were described using parameters of the LEMON law (look external, evaluate 3–3-2 rule, Mallampati score [21], obstruction, immobilisation). Degree of visualization of the vocal cords was described using Cormack/Lehane (C/L) grade [22, 23] as assessed by direct or video laryngoscopy. The intubations’ difficulty scale (IDS) was calculated for each patient [24]. A difficult intubation was defined as one that requires more than two attempts or an IDS ≥5 points [24].

For ED airway management, intubation conditions (very good = glottis open, good = glottis open and less combative patient, poor = glottis nearly closed and combative patient, very bad = glottis closed) were recorded. Moreover, any complication during RR airway management was documented. Complications (e.g. oxygen desaturation, hypotension) were defined in accordance to Sakles et al. [6].

### Statistical analysis

Data were entered into Microsoft Excel 2014 (Microsoft, Germany) and analysed using SPSS (IBM-Statistics, Version 20, IBM Inc., Armonk, NY, USA). Descriptive statistics included number or percentages, mean (SD), median and minimal to maximal value. Chi^2^-test or, as appropriate, Fisher’s exact test were used to compare groups of binary data and to test for trends. For all analyses, actual *P*-values were reported and all tests were two-tailed. Statistically significant differences were considered at *p* < 0.05 level.

## Results

During the 12-month study period, 34,303 patients were admitted to the ED. 13,229 patients with 592 treated in the RR were excluded due to trauma as leading cause of admission. 21,074 patients were admitted for non-traumatic emergencies, with 537 patients directly admitted to the RR (2.54%). After excluding five patients due to incomplete datasets, 286 critically ill non-traumatic patients receiving airway management in the RR were further investigated (53.8%).

**Fig. 1:**
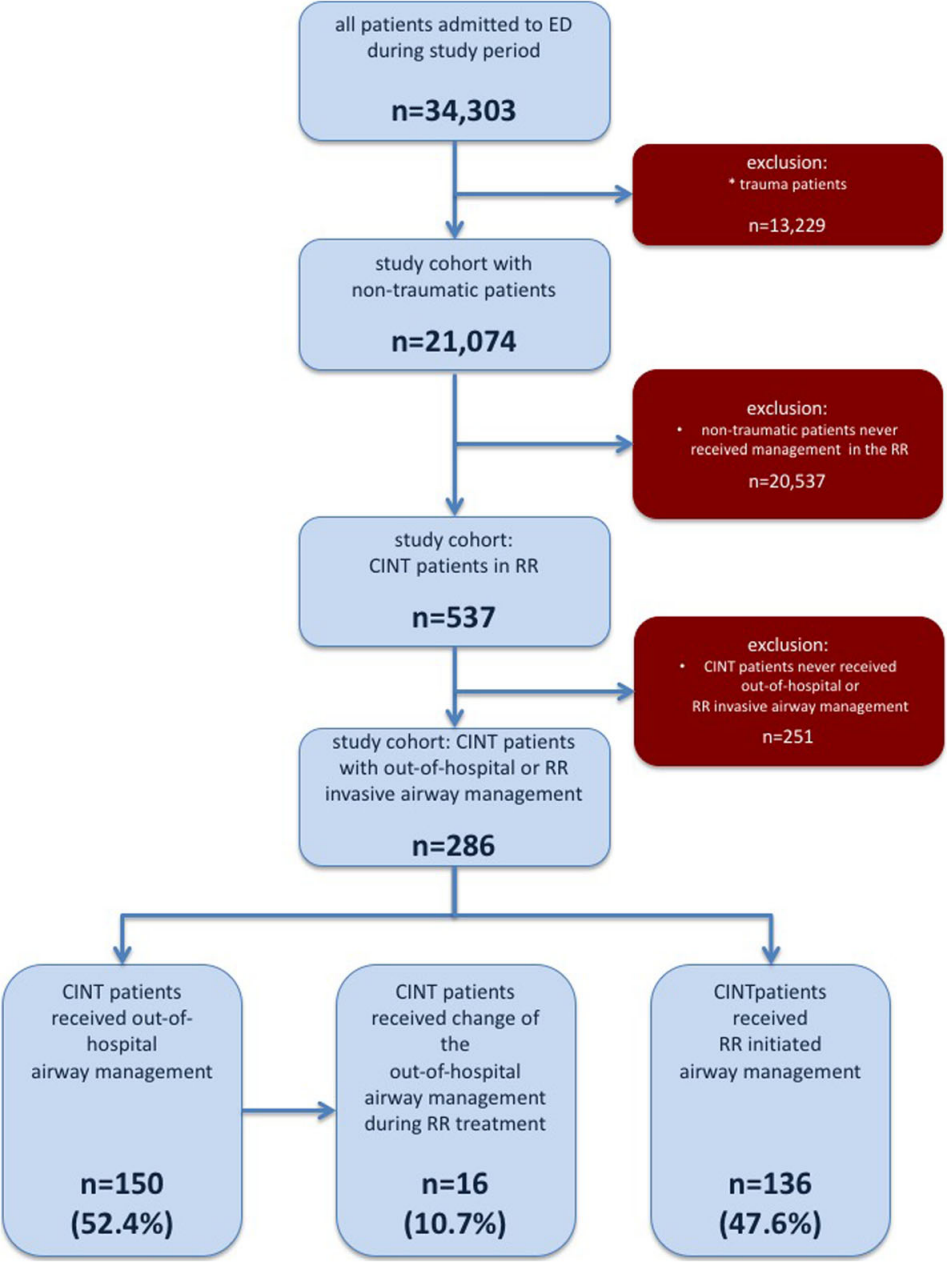
Study cohort: ED = emergency department, CINT = critically ill non-traumatic, RR = resuscitation room

In 150 (52.4%) patients, airway management was performed by EMS before and in 136 (47.6%) patients by ED staff after admission to the RR (Fig. 1). In 11 (7.3%) patients of the EMS group, the airway was secured with a laryngeal tube by paramedics. In 7 out of these 11 (63.6%) cases, an EMS physician had changed the airway device into an endotracheal tube in the out-of-hospital setting. In 16 (10.7%) patients of the EMS group, the airway device had to be changed after RR admission due to various reasons. The patient characteristics in the EMS and the RR management group were comparable (Table 1). However, according to the out-of-hospital triage score, patients with out-of-hospital airway management had a higher NACA (5.3 ± 0.8 vs. 4.8 ± 0.7, *p* = 0.001) and ASA score (3.5 ± 1.3 vs. 3.2 ± 0.9, *p* = 0.007) in comparison to patients with in-hospital airway management in the RR. The leading indication for airway management in the field and the RR setting differ significantly, with cardiac arrest in the out-of-hospital setting and unconsciousness as well as respiratory failure in the RR setting (Table 1).

**Table 1:** Patient’s characteristics

	out-of-hospital airway management (*n* = 150)	ED airway management (*n* = 136)	p
Epidemiology			
age (years), MV ± SD,	66 ± 16	65 ± 18	0.730
Median, min-max	69, 18–94	71, 20–89	
Weight (kg), MV ± SD,	83 ± 27	81 ± 22	0.403
Median, min-max	80, 42–180	80, 40–150	
Hight (cm), MV ± SD,	170 ± 32	170 ± 9	0.992
Median, min-max	170, 150–190	170, 140–190	
BMI (kg/m^2^), MV ± SD,	28 ± 8	28 ± 7	0.419
Median, min-max	28, 15–58	26, 16–59	
Male Gender [n, (%)]	86 (57.3%)	82 (60.2%)	0.611
NACA (points), MV ± SD,	5.3 ± 0.8	4.8 ± 0.7	***0.001***
Median, min-max	5.5, 3–6	5, 3–6	
ASA (points), MV ± SD,	3.5 ± 1.3	3.2 ± 0.9	***0.007***
Median, min-max	4, 1–6	3, 1–5	
Reason for airway management			
Cardiac arrest [n, (%)]	74 (49.3%)	9 (6.6%)	***< 0.001***
Unconsciousness [n, (%)]	50 (33.3%)	58 (42.6%)	0.105
Respiratory failure [n, (%)]	18 (12.0%)	50 (36.8%)	***< 0.001***
Hemodynamic instability [n, (%)]	8 (5.3%)	19 (14.0%)	***0.01***

### Patients with out-of-hospital airway management in the resuscitation room

Patients who received airway management by EMS physicians (*n* = 150) underwent endotracheal intubation or laryngeal tube insertion in 90.7% (*n* = 136) and 9.3% (*n* = 14), respectively. Out of hospital capnography was used in 82.7%. Oesophageal intubation was detected in two cases (1.5%) of the out-of-hospital intubation group. In one of these patients capnography had not been used in the field or during transport. Both patients were admitted with on-going cardiopulmonary resuscitation and ED physicians secured the airway within the first intubation attempt using direct laryngoscopy (each C/L grade 1). In both cases, there were no predicted or occurred difficult airways using LEMON law and IDS score.

In the 14 patients with out-of-hospital inserted laryngeal tubes, we observed insufficient ventilation (e.g. airway leakage) in 8 cases (57.1%), in 75% without out-of-hospital use of capnography. During the RR period, all 14 patients with laryngeal tube were successfully intubated using direct vs. video laryngoscopy (42.9%, *n* = 6 vs. 57.1%, *n* = 8) within 1.3 ± 0.5 (Median: 1, min-max 1–2) vs. 1.9 ± 1.4 attempts (Median: 1.5, min-max 1–5), respectively. We did not observe a significant difference according to LEMON law (0.7 ± 0.5 vs. 0.6 ± 0.5 points) or IDS score (2.7 ± 0.5 vs. 2.0 ± 1.9) comparing direct vs. video laryngoscopy, while C/L grades were significantly different (2.3 ± 1.0 vs. 1.4 ± 0.5, *p* = 0.04).

### RR patients without out-of-hospital airway management

In 136 patients, airway management was initiated first after RR admission. A tracheotomy tube change was necessary in 2 cases, both successful at the first attempt. The other patient had been intubated with first-pass, second-pass, and third-pass intubation success rates of 70.9% (*n* = 95), 14.9% (*n* = 20), and 0.8% (*n* = 1), respectively. Overall, 100% of the intubations were successful in mean after 1.3 ± 0.8 intubation attempts (Median: 1, min-max: 1–6). Multiple intubation attempts were needed in 39 cases (29.1%). The intubation procedure was handed over to another physician in 14 cases (10.4%), as required by the institutional ED safety protocol. In the cases handed over, 1.2 ± 0.4 intubations attempts were required for successful intubation by the next provider (Median 1, min-max: 1–2).

Direct laryngoscopy and video laryngoscopy was used in 69.9% (*n* = 94) and 30.1% (*n* = 40), respectively. Overall, the needed mean number of intubation attempts in the direct (macintosh blade) and video laryngoscopy (macintosh-like blade) group with 1.2 ± 0.5 vs. 1.2 ± 0.4 were comparable (*p* = 0.887).

The percentage of anticipated difficult airways estimated by the acting physician was 23.5%. The prediction of difficult airways according to patients with at least one positive LEMON criterion and with an IDS ≥5 points was 37.5 and 11.6%. The difficult airway characteristics of the patients are presented in Table 2, and the difficulties contributed to problems during RR intubation procedures were shown in Table 3.

**Table 2:** Difficult airway characteristics (n = 136)

	[n, (%)]
anticipated difficult airway	32 (23.5%)
LEMON	
0 points	85 (62.5%)
LEMON ≥1 point	51 (37.5%)
IDS	
0 points	39 (28.8%)
1–5 points	81 (59.6%)
≥ 5 points	16 (11.6%)
Cormack/Lehane I	59 (43.4%)
II	40 (29.4%)
III	23 (16.9%)
IV	4 (2.9%)
not documented	10 (7.4%)*

*including 2 patients with tracheotomy tube exchange

**Table 3:** Difficulties contributed to problems during resuscitation room intubation procedures (*n* = 129)

	[n, (%)]
Secretion/blood	21 (16.3%)
Reduced mouth opening	12 (9.3%)
Short neck	9 (8.5%)
Immobilisation	7 (5.4%)
Untrained personal	7 (5.4%)
Retrognathy	4 (3.1%)
Patient positioning	3 (2.3%)
Anatomy pharynx/larynx	3 (2.3%)
Foreign body	1 (1.6%)
Anatomy neck	0 (0.0%)
Malfunction equipment	0 (0.0%)

BUHE and supine, as patient positioning for endotracheal intubation, were used in 50.7% (*n* = 68) and 44.8% (*n* = 60), respectively. In order to optimize the first intubation attempt, stylets, NBA, Jackson’s position, BURP, and suction units were used in 91.0% (*n* = 122), 82.1% (*n* = 110), 70.9% (*n* = 95), 26.9% (*n* = 36), and 14.2% (*n* = 19).

**Fig. 2:**
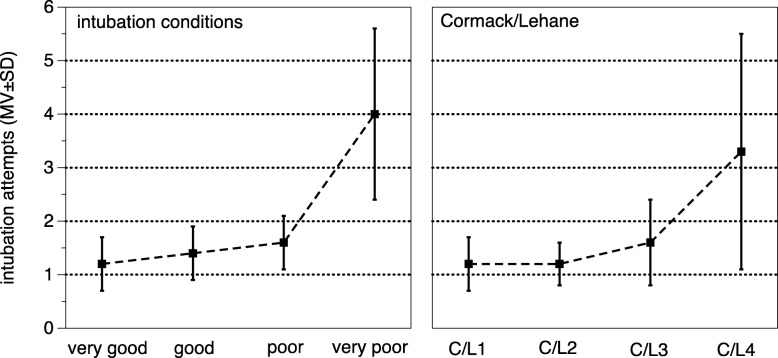
Number of mean intubations attempts according to intubations conditions and Cormack/Lehane grade. MV = mean value, SD = standard deviation

The mean number of needed intubation attempts correlated with the intubation condition categories “very good/good” and “bad/very bad” with 1.2 ± 0.5 vs. 2.2 ± 1.4 (*p* = 0.0001) and C/L grade 1/2 and 3/4 (1.2 ± 0.5 vs. 1.8 ± 1.2, *p* = 0.0002) (Fig. 2). First-pass success was associated with C/L 1, 2, 3 and 4 with 79.5, 77.5, 56.5, and 25.0%, respectively. Patient positioning in BUHE or supine did not affect the C/L grade (BUHE vs. supine: C/L grade 1/2: 78.1 vs. 79.3%; C/L grade 3/4: 21.9 vs. 20.7, *p* = 0.873). Direct laryngoscopy compared with video laryngoscopy did not lead to better C/L grade 1/2 (81.3 vs. 73.9%, *p* = 0.334).

Complications and adverse events were documented in 129 out of 136 patients (94.9%). The most common complications and adverse events during RR airway management were hypotension (20.4%) and desaturation (9.3%) (Table 4**)**. The overall complication rate was 42.6%. The complication rates (and mean number of intubations attempts) increased according to the C/L grade 1, 2, 3 and 4 as following 24% (1.2 ± 0.5), 25% (1.2 ± 0.4), 24% (1.6 ± 0.8), and 75% (3.3 ± 2.2).

**Table 4:** Complications during airway management in resuscitation room (n = 129)

Hypotension (decrease in SBP to < 90 mmHg)	26 (20.2%)
desaturation (decrease in oxygen saturation ≥ 10%)	12 (9.3%)
oesphageal intubation	7 (5.4%)
aspiration	4 (3.1%)
endobronchial intubation	2 (1.6%)
cardiac arrest	4 (3.1%)
complications	55 (42.6%)

*SBP* systolic blood pressure

## Discussion

This prospective single centre study evaluated the out-of-hospital and ED initiated airway management in adult non-traumatic critically ill patients in an academic German ED during a one-year observational period. The primary goal was to evaluate the out-of-hospital airway management performed by EMS physicians at hospital arrival and to document the airway management in the RR setting in the ED in order to describe incidence, airway technique, success and complication rates.

Several ED airway registries exist worldwide (e.g., Australia [8], North America [9, 10], Korea [11], Japan [12]), however data on emergency airway management in German EDs are still missing. Thereby, the introduction of an airway registry is an important issue for quality assurance [25]. To our knowledge, this is the first study investigating RR airway management in non- traumatic patients in a German ED setting.

In patients with out-of-hospital airway management admitted to the RR we found a low incidence of oesophageal intubation with 1.5% in comparison to other studies that reported a rate of 5.1–6.7% in German physician-staffed EMS [26, 27]. Interestingly, 9.3% of the admitted patients were treated with a laryngeal tube. According to institutional policy all 14 patients with SADs were intubated immediately after RR admission. In 57% of these SAD patients ventilation was insufficient at RR admission. Comparable complications and adverse events rates after out-of-hospital laryngeal tube insertion were also reported elsewhere [28–30]. One of the major concerns is that only 82.7% of patients received capnography in the out-of-hospital setting. Oesophageal intubations, as well as insufficient ventilation after insertion of a laryngeal tube would likely be recognized during the out-of-hospital airway management if capnography would solely have been used [28, 31].

As described in the study protocol only critically ill non-traumatic patients were investigated in this study and patients with trauma were excluded. However, this study population may restrict the comparability of our results to other airway registries [8, 10, 32, 33].

In the second part of this investigation, the observed sample size of 136 ED initiated airway procedures in our ED is comparable with those in other large ED airway registries (including 50–90 cases per year) [10, 34]. In addition, we investigated RR patients with out-of-hospital airway management already performed by EMS physicians. However, 16 of theses cases with insufficient ventilation and oxygenation needed immediate airway management after hospital arrival. The observed first-pass success rate of the 134 patients receiving invasive airway management after RR admission was 70.9%. These findings were in line with previous analysis of ED airway registry reporting a first-pass success range of 61–94% [7, 8, 10, 33–35]. However, the first-pass success rate in this study was lower than in the meta-analysis by Park et al. [36] founded 84% as an ED benchmark. The aim of improving first-pass success should be paramount since it is well known that multiple intubation attempts are associated with significant increases in complications [6, 7, 12]. The overall airway management success in this investigation was 100% and comparable with the results of other airway registries and ED studies [7, 10, 32, 34]. Overall, the airway of all patients was secured using endotracheal intubation, excluding two patients with tracheostomy tube change (1.5%). Contrary to other investigations [10, 25, 32], fiberoptic intubation and cricothyroidotomy was not performed during the study period. However, with an anticipated incidence of cricothyroidotomy of 0.3%, it is likely only a question of time for this procedure to also be seen in our institution.

The intubation procedure was performed in two-thirds of cases using direct laryngoscopy with Macintosh blades, and less often using C-MAC video laryngoscopes with Macintosh-like blades. Other investigations found a comparable rate of video laryngoscopy use in 39–48% [8, 34]. It is anticipated that the incidence of video laryngoscopy assisted intubation will increase in the upcoming years [10, 33].

A difficult airway was anticipated in 23.5% of patients receiving RR airway management. One-third of airways were predicted as difficult per LEMON law, and a moderate to severe intubation situation was observed in 11.6% per IDS. These findings were in the range with data reported from other airway registries [33]. In line with previous investigations, problems associated with difficulties during ED airway management were most often secretion or blood in the pharynx, reduced mouth opening, short neck and immobilization [4]. In contrast to Khandelwal et al. [20] and Turner et al. [37], we did not find an association between C/L grade and BUHE or supine position in ED airway management. Hossfeld et al. [38] reported an improved visualization using video laryngoscopes (with Macintosh-like blade) compared to standard Macintosh laryngoscopes. However, in line with some investigations [39], we found similar C/L grade 1/2 using video laryngoscopes in comparison to direct laryngoscopy with standard Macintosh blade.

Complications associated with the intubation procedures were observed in 42.6%. Other studies reported complication rates between 10 and 29% [8, 32–34]. Differences in the reported complication rates are at least in part due to varying definitions of complications in other airway registries. Hypotension was the most common reported complication with 20%, which is in line with other investigations reporting an incidence of 7–18% [40, 41]. The incidence of immediately detected and corrected oesophageal intubations in 5.4% was in line with other ED studies [8, 34]. Immediate recognition of oesophageal intubation using capnography is imperative to prevent hypoxemia [31]. In the RR, we used capnography without exception. Desaturation occurred in this study with 9.3% and which is comparable to other out-of-hospital and ED airway registries (11–16%) [8, 33, 42].

As a limitation of this study, we need to mention that we performed but did not document specific procedures for preoxygenation (e.g., delayed sequence intubation using non-invasive ventilation for preoxygenation [43]) or apnoeic oxygenation [44]. Including these procedures to further study protocols seems to be necessary. Moreover, the kind of laryngoscopy (video vs. direct laryngoscopy) should be documented in further studies. Cardiac arrest as a major complication during ED airway management occurred in the present investigation at a rate of 3.1%, which was comparable to other out-of-hospital and ED investigations with a reported range between 1.5–4.4% [8, 34, 45].

Rapid sequence induction using neuromuscular blocking agents was performed in 87.5% in the RR setting. These findings are in line with other data from ED airway registries described percentages between 73 and 92% [10, 25, 32, 34]. However, there are other data from a Japanese ED airway registry stated a lower rate of RSI use with only 20% [35]. Comparable with other investigations [34], the most frequent used neuromuscular blocking agent was rocuronium in 85%.

Taking together, the game changer in out-of-hospital airway management are preoxygenation (e.g. delayed sequence intubation), using of video laryngoscopy and muscle relaxation [43, 46].

Our study suffers from several limitations. At first, this study was carried out at a single institution and so the results cannot be taken to be representative of all EDs in Germany, or other places in the world. Nevertheless, this study provided detailed information about German RR airway management in critically ill non-traumatic patients for the very first time. Furthermore, the study was observational in nature, neither randomized nor controlled. The team leader was required to complete the airway registry form. Reporter bias is difficult to exclude, and there may be a tendency to document an improved glottis visualisation and underreport complications. The self-developed emergency airway registry form was combined with the information of medical charts, which has been reported to be beneficial [45]. The team leaders were instructed repeatedly and attempts to improve accuracy were made by interviewing the ED physicians and by reviewing the medical record.

Due to the fact that in Germany a multi-centre airway registry does not exist, we suggest that this should be initiated in order to analyse the situation countrywide. Studies identified more than eleven emergency airway registries that sometimes widely differed concerning inclusion period, inclusion criteria, definition of complications and application of newer methods of emergency airway management [47]. Comparability of the reported results and first-pass-success rates is only possible to a limited extent. Therefore, standardised reporting forms should be used in order to make the results comparable. Using the data, benchmarking would be possible, with systematic investigation on first-pass success, techniques, complications and adverse events. Moreover, the effect of new techniques in the ED setting concerning emergency airway management over the years will be detectable as described by Brown et al. [34]. Using these data, procedural and structural optimisation of this important field will be possible.

## Conclusions

In conclusion, RR airway management of critically ill non-traumatic patients has substantial challenges. Our study results confirm that RR airway management is a high-risk procedure. We propose a nation-wide airway registry to better track outcomes of RR airway management in the future.

## Additional file

Additional file 1: Resuscitation Room Admission criteria for non-traumatic critically ill patients according to the ABCDE approach^a,b^. (DOCX 15 kb)

## Abbreviations

AM: Airway management
ASA: American Society of Anaesthesiology
BUHE: Back-up head elevated or supine position
C/L: Cormack/Lehane score
CINT: Critically ill non-traumatic patients
ED: Emergency department
EMS: Emergency medical service (in Germany with emergency doctors and emergency paramedics)
IDS: Intubations’ difficulty scale
LEMON: Look external, evaluate 3–2-2 rule, Malampati score, obstruction, immobilisation
NACA: National Advisory Committee of Aeronautics
OcEAN: Observation of airway management in Emergency Department
RR: Resuscitation room
SAD: Supraglottic airway device
