# Supplementary material for: Airway Management in the Emergency Department (The OcEAN-Study) - a prospective single centre observational cohort study
Source: Scand J Trauma Resusc Emerg Med. 2019 Feb 14;27:20. doi: 10.1186/s13049-019-0599-1 (PMC6376794; doi:10.1186/s13049-019-0599-1)
Supplement: Supplementary file 1 — Resuscitation Room Admission criteria for non-traumatic critically ill patients according to the ABCDE approacha,b. (DOCX 15 kb) [file 13049_2019_599_MOESM1_ESM.docx]

**SUPPLEMENTAL MATERIAL**

**Additional file 1: Resuscitation Room Admission criteria for non-traumatic critically ill patients according to the ABCDE approach^a,b^**

**problem example**

A airway enoral swelling (e.g. angiooedema, haematoma of the tongue, allergic reaction, abscess), free airway at risk
 out-of-hospital airway management (including all alternative
 devices)

B breathing respiratory insufficiency with high respiratory rate (suggested
/ventilation rapid deterioration) and
 low oxygenation level using pulse oximetry

Patients requiring rapid airway management (announcement of
 difficult airway)
 patients under non-invasive and mechanical ventilation

C circulation circulatory insufficiency (e.g. hypotension, shock of each
 origin),
 after and ongoing cardiopulmonary resuscitation

relevant cardiac arrhythmia (e.g. AV block III°, ventricular
 tachycardia, ventricular fibrillation)
 bleeding (e.g. esophageal varices)

D disability relevant altered mental state

intracerebral bleeding/stroke and other with ABCDE problem

E Environment intoxication with ABCDE problem
 other critical hypothermia
 conditions hyperthermia

^a^senior ED physician in charge decides to activate the emergency department resuscitation room
^b^the acitivation of resuscitation room depends only on patient risk not from EMS with or without EMS physician
